# Investigation of the Earliest Ozone Pollution Events in Hangzhou Bay, China Based on Observations and ERA5 Reanalysis Data

**DOI:** 10.3390/toxics13020099

**Published:** 2025-01-27

**Authors:** Tianen Yao, Xinhao Li, Zhi Li, Xinyu Yang, Jinjia Zhang, Yaqi Wang, Jianhui Guo, Jing Li

**Affiliations:** 1 Department of Global Health, School of Public Health, Peking University, Beijing 100191, China; kallen@stu.pku.edu.cn (T.Y.); 2022312336@email.cufe.edu.cn (X.Y.); 2 Institute of Child and Adolescent Health, School of Public Health, Peking University, Beijing 100191, China2211110227@bjmu.edu.cn (Y.W.); 2311110213@bjmu.edu.cn (J.G.); 3 Georgia Institute of Technology, School of Earth and Atmospheric Sciences, Atlanta, GA 30332, USA; xli3111@gatech.edu; 4 Dongsheng District Meteorological Service, Ordos 017099, China; 5 National School of Development, Peking University, Beijing 100871, China; jinjiazhang@stu.pku.edu.cn

**Keywords:** ozone, Hangzhou Bay, NO_x_-limited, solar radiation

## Abstract

Ozone pollution in Hangzhou Bay, one of the seven petrochemical clusters in China, is severe. Early ozone pollution has been detected recently, such as the maximum daily 8 h average (MDA8) ozone concentration in Jiaxing achieving 171.0 μg/m^3^ on 7 March 2023. Satellites have observed tropospheric column concentrations of ozone precursors formaldehyde (HCHO) and nitrogen dioxide (NO_x_), and quantitative models are proposed to reveal the causes of the early onset of ozone pollution. VOC-limited and transitional regimes dominate most areas in Hangzhou Bay, and NO_x_-limited regimes dominate the region around Hangzhou Bay, such as northeastern Jiangsu Province. Results show that HCHO column concentrations are increasing in VOC-limited regions, and NO_x_ column concentrations are increasing more rapidly than HCHO in NO_x_-limited regions. According to multivariate linear regression (MLR), early spring ozone pollution in Hangzhou Bay is mainly caused by meteorological drivers. Hangzhou Bay has formed an atmospheric meteorological environment with high temperature and low humidity. The richer solar radiation intensifies the photochemical reactions associated with tropospheric ozone formation, producing more tropospheric ozone. Based on the Shapley Additive Explanation (SHAP) algorithm, ozone pollution increases when solar radiation exceeds 12 million J/m^2^ and is accompanied by high temperatures. Overall, reducing VOC emissions helps to mitigate ozone growth in Shanghai and northern Hangzhou Bay, while reducing NO_x_ emissions is more effective in northeastern Jiangsu Province.

## 1. Introduction

Ozone pollution is a prominent and major environmental issue in the metropolises of China [1,2,3]. Specifically, as a potent oxidant and greenhouse gas, tropospheric ozone has shown a steady increase, with ozone pollution occurring earlier each year [4,5]. This atmospheric oxidant can induce negative impacts on human health, such as in respiratory and cardiovascular abilities, and on surface vegetation, including agricultural crops [6,7,8]. The Hangzhou Bay region, as one of China’s largest petrochemical clusters, suffers from severe ozone pollution due to industrial concentration and its exposure to land–sea breezes [9,10]. This high ozone level could intensify global warming, potentially creating a positive feedback loop leading to more frequent pollution events [3,11]. Thus, it is urgent that we reveal the underlying drivers of ozone generation in early spring in the Hangzhou Bay region and prevent this generation through more comprehensive monitoring and regulatory measures.

The formation of ozone pollution is usually attributed to anthropogenic and meteorological factors [12,13]. Most ozone cannot be generated spontaneously within the troposphere as a secondary pollutant [14]. Therefore, its occurrence should be studied in terms of its several precursors, mainly associated with excessive emissions from industrial activities and transportation [13,15]. As two primary chemical precursors of ozone, nitrogen oxides (NO_x_) and volatile organic compounds (VOCs) could engage in photochemical reactions resulting in tropospheric ozone formation and contribute to ozone pollution processes [16,17]. Due to the non-linear relationship between ozone and its precursors [16,18], understanding the formation of the tropospheric ozone requires examining the complex interactions within ozone–NO_x_–VOC chemistry [19,20].

Despite the impact of human activities, the contribution of natural factors cannot be neglected, as atmospheric conditions could promote the formation of tropospheric ozone [11]. In the Yangtze River Delta (YRD) region where Hangzhou Bay is located, ozone level presents clear seasonal patterns, typically peaking in warm seasons from May to July [21]. Therefore, previous studies have primarily focused on ozone pollution during the summer months, often overlooking pollution events occurring outside of this peak season [4].

Our study was initiated by an ozone pollution incident that occurred in early March 2023. According to Shanghai Environmental Monitoring Center, the air quality index (AQI) in Shanghai reached 102 on 10 March, indicating mild air pollution. The main atmospheric pollutant of this event was ozone. The maximum daily 8 h average (MDA8) ozone concentration in Shanghai was recorded as 162 μg/m^3^, surpassing typical spring ozone levels and positioning this as the earliest recorded ozone pollution event in the last decade. This anomaly raises questions regarding the underlying causes, particularly considering the area’s unique geographic and industrial profile—Hangzhou Bay hosts a dense distribution of industrial parks, which emit high levels of ozone precursors such as nitrogen oxides and VOCs [5,10].

The limited research on springtime ozone pollution, particularly in the context of earlier seasonal onset, underscores a gap in understanding the contributing factors to unusual ozone events. Therefore, we aim to (1) reveal the temporal and spatial distribution of the early occurrence of pollution in Hangzhou Bay, (2) employ ERA5 meteorological data for detecting the meteorological effects on ozone pollution, (3) reveal the impact of ozone precursors based on ozone–NO_x_–VOC sensitivities, and (4) quantify the contributions of meteorological and anthropogenic factors to spring ozone pollution.

## 2. Data and Methods

### 2.1. Study Area

**Figure 1:**
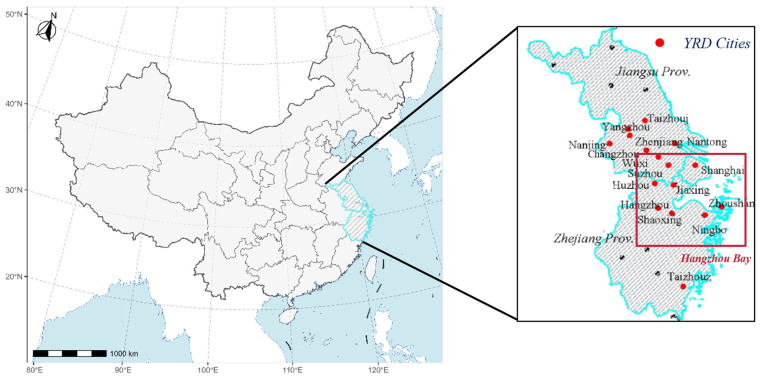
The location of the Hangzhou Bay and Yangtze River Delta (YRD) region in China.

The Hangzhou Bay (28.9° N–31.7° N, 119.8° E–123° E), located in the YRD region, encompasses 98 monitoring sites and several major cities, including Shanghai, Hangzhou, Ningbo, Jiaxing, Huzhou, Shaoxing, and Zhoushan (Figure 1). From 2019 to 2023, the Hangzhou Bay region, as a petrochemical industry cluster and a metropolitan agglomeration, has presented high VOC and NO_x_ emission intensity resulting in severe ozone pollution [20]. Besides, the data in this paper was processed by python 3.10, R 4.2.1, and MATLAB 2023b.

### 2.2. Near-Surface Observations of Ozone

To consider the entire process of this earliest ozone pollution event and compare it with ozone concentrations at the same time in previous years comprehensively, a study period was determined between 1 March and 10 March of each year from 2019 to 2023. Ozone data between 1 March and 10 March during 2019–2023 were studied for all observational sites and cities in the Hangzhou Bay region. Ozone pollution in the second half of March 2023 was therefore not included in the study. According to the technical regulation for ambient air quality assessment, the daily maximum 8 h average (MDA8) ozone concentration (μg/m^3^) could be an indicator for the daily ozone pollution evaluation, and the MDA8 ozone concentration was calculated based on ozone 8 h datasets from the National Urban Air Quality Real-Time Publishing Platform (https://air.cnemc.cn:18007/, accessed on 15 April 2023). All the ozone-relevant data provided by this platform were recorded hourly, thus supporting the analysis of the ozone pollution situation in the Hangzhou Bay region.

The comparison of the earliest occurrence date of the ozone pollution event in 2023 with that of previous years can be achieved through MDA8 ozone datasets from observational cities. The distribution of MDA8 ozone concentrations between 1 and 10 March can be interpreted by MDA8 ozone datasets from observational sites, which could also facilitate the study of the ozone concentration evolution in the Hangzhou Bay region during 2019–2023.

### 2.3. Satellite Observations of NO_x_ and VOCs

NO_x_ and VOCs are important tropospheric ozone precursors. Typically, NO_x_ can be referred as the combination of nitrogen oxide (NO) and nitrogen dioxide (NO_2_) [22]. The interconversion between NO and NO_2_ involved in the ozone photochemical cycle could occur within a few minutes, which means that NO_2_ could be considered as a proxy for NO_x_ [23]. In addition, formaldehyde (HCHO) is an intermediate gas in almost all oxidation reactions of non-methane volatile organic compounds (NMVOCs). Hence, the concentration of HCHO can be attributed to the levels of VOCs [24].

NO_x_ and VOC data were obtained from Sentinel-5P TROPOMI observations (https://developers.google.com/s/results?q=Sentinel-5P%20TROPOMI%20observations%20&text=Sentinel-5P%20TROPOMI%20observations, accessed on 10 May 2023), which provide datasets beneficial for assessing the air quality, such as NO_2_ and HCHO column concentrations. TROPOMI datasets (5 × 3.5 km^2^ for the spatial solution) are available daily from 2019 until the present, which covers the whole study period (1–10 March) between 2019 and 2023. Furthermore, TROPOMI datasets for two precursors in the Hangzhou Bay area were averaged from 1 to 10 March of each year to investigate this ozone pollution event. In detail, the NO_x_ concentration corresponds to the tropospheric NO_2_ column number density (mol/m^2^) of TROPOMI datasets, representing the tropospheric vertical column of NO_2_. The VOC concentration refers to the tropospheric HCHO column number density (mol/m^2^) of TROPOMI datasets, which indicates the tropospheric vertical column of HCHO.

### 2.4. Ozone–NO_x_–VOC Sensitivity in the Hangzhou Bay Region

To gain an accurate understanding of the impact of these two precursors on the formation of tropospheric ozone, an indicator has been proposed to facilitate the analysis of the causes of ozone pollution in different scenarios, since the relationship between primary and secondary pollutants is highly non-linear [18,25]. The HCHO/NO_2_ ratio has been identified as a potential indicator for the tropospheric ozone–NO_x_–VOC sensitivity, because the HCHO/NO_2_ column ratio is approximately equivalent to the VOC/NO_x_ ratio [26]. In addition, the threshold values of their ratio can be specific for the transition between VOC-limited (or NO_x_-saturated) and NO_x_-limited (or NO_x_-sensitive) regimes under certain spatial circumstances [18,26].

With TROPOMI datasets of NO_2_ and HCHO column number densities, the HCHO/NO_2_ threshold values could be calculated for the Hangzhou Bay region under different regimes [27]. Previous literature has suggested that the threshold values of HCHO/NO_2_ for the Hangzhou Bay region (included in the Shanghai–Jiangsu–Zhejiang–Anhui region) were 2.2 and 3.3, which can divide the Hangzhou Bay into three ranges: less than 2.2, between 2.2 and 3.3, and greater than 3.3 [20]. In this case, the mechanisms by which NO_2_ and HCHO trigger the earliest onset of this ozone pollution event would be more intuitively resolved by analyzing different regimes of ozone–NO_x_–VOC chemistry in Hangzhou Bay.

### 2.5. Quantitative Models

#### 2.5.1. Multivariate Linear Regression

ERA5 reanalysis data (https://cds.climate.copernicus.eu, accessed on 4 May 2023) with a spatial resolution of 0.25° × 0.25° provide hourly measurements of air temperature (at 1000 hPa), surface downward solar radiation flux, temperature, boundary layer height (BLH), pressure, relative humidity (RH), total cloud cover, wind direction, wind speed, and precipitation [28]. We use detrend MDA8 ozone, obtained by removing the average of 1 to 10 March each year from 2019 to 2023 in 98 monitoring sites. Multivariate linear regression (MLR) is usually proposed to quantify the role of meteorological factors in ozone formation. We employ the ozone difference fitted by MLR to profile meteorological trends, which can be expressed as (1):

(1) $$M_{m}=\sum_{i=1}^{10}\beta_{i}X_{i}+a+e\;$$

where $M_{m}$ is the fitted ozone value with meteorological factors, *X* denotes every weather pattern from ERA5, β is the regression coefficient, *a* is the intercept term, and *e* is the residual.

The residuals in Equation (2) are part of the anomaly not explained by the MLR and are defined as the anthropogenic effects of ozone generation in Hangzhou Bay. Moreover, the presumed anthropogenic-driven ozone formation (*M_r_*) can be expressed as:

(2) $$M_{r}=M_{o}-M_{m}\;$$

where $M_{o}$ is the observed MDA8 ozone concentration in Hangzhou Bay.

#### 2.5.2. Shapley Additive Explanation

MLR, as a traditional statistical model, usually has rigorous assumptions such as normal distribution and covariance problems. Machine learning usually has high fitting ability and is good at dealing with non-linear problems, such as through the Light Gradient Machine (LightGBM). We apply LightGBM to fit the meteorological factors and then further use the Shapley Additive Explanation (SHAP) algorithm to quantify and visualize the contribution of the meteorological factors to ozone pollution in Hangzhou Bay and explain the interactive effects between meteorological factors. The SHAP value for each meteorological factor is [29]:

(3) $$\begin{array}{c} {MDA8}_{i}={MDA8}_{i(base)}+\sum_{j=1}^{S}shap(x_{i,j}) \end{array}$$

(4) $$\begin{array}{c} shap\left(x_{i,j}\right)=\sum_{M}\frac{\left|M\right|!\left(S-\left|M\right|-1\right)!}{S!}{(MDA8}_{\left(M\cup \{x_{i,j}\}\right)}-{MDA8}_{\left(M\right)}) \end{array}$$

where ${MDA8}_{i(base)}$ indicates the expectancy value of ${MDA8}_{i}$, *shap*($x_{i,j})$ is the SHAP value of the contribution of factor *j* to *MDA8_i_* at moment *i*, *S* is the set of all input features, x denotes the meteorological drivers of MDA8 ozone from ERA5, and *M* presents the subset of features excluding factor *j*.

Furthermore, the SHAP technique can reveal the interaction effects between the meteorological drivers by the dependence plot [30]. The interaction based on the SHAP values can be expressed as [31]:

(5) $$shap\left(x_{i},x_{j}\right)=\sum_{M^{*}}\theta \delta_{i,j}(P^{*})$$

(6) $$\theta =\frac{\left|P^{*}\right|!\left(S-\left|P^{*}\right|-2\right)!}{2\left(S-1\right)!}$$

(7) $$\delta_{i,j}\left(P^{*}\right)={(MDA8}_{\left(P^{*}\cup \{x_{i},x_{j}\}\right)}-{MDA8}_{\left(P^{*}\cup \left\{x_{j}\right\}\right)})-{(MDA8}_{\left(P^{*}\cup \left\{x_{i}\right\}\right)}-{MDA8}_{\left(P^{*}\right)})$$

where $shap\left(x_{i},x_{j}\right)$ is the interaction value between factors *i* and *j*, ${MDA8}_{\left(M^{*}\right)}$ indicates the simulated value of the machine learning algorithm under subset P*, P* denotes the subset of features excluding factors *i* and *j*, and θ depicts the weight of the subset of features P*.

## 3. Results

### 3.1. MDA8 Ozone Pollution in Hangzhou Bay

#### 3.1.1. A Noticeable Earliest Ozone Pollution Event

The MDA8 ozone concentrations and dates of the earliest ozone pollution events in Hangzhou Bay for each year between 2015 and 2023 are summarized in Table 1. All events occurred in spring, predominantly in March. Notably, the earliest ozone pollution event in 2023 in the Hangzhou Bay region occurred on 7 March, which is earlier than the earliest ozone pollution events in previous years. This earliest ozone pollution event happened in Jiaxing, even ahead of Shanghai. The MDA8 ozone concentration in Jiaxing was up to 171.0 μg/m^3^.

Over time, these earliest ozone pollution events have shifted towards earlier dates with relatively lower pollutant concentrations, in contrast to the initial two years (2015–2016), where events occurred later alongside higher pollutant concentrations. In addition, while the earliest ozone pollution events were concentrated in Huzhou during the first three years, they gradually dispersed throughout different cities. However, no earliest ozone pollution events were recorded in Ningbo or Zhoushan for the Hangzhou Bay region throughout the entire 2015–2023 period.

#### 3.1.2. Spatial Distribution and Variation of MDA8 Ozone During 2019–2023

**Figure 2:**
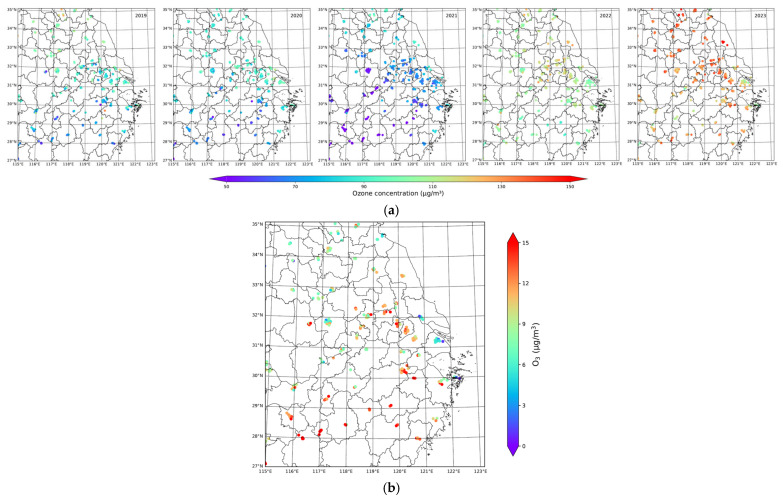
Average of MDA8 ozone concentrations between 1 March and 10 March (2019–2023) at sites in the Hangzhou Bay region and their varying trends. (**a**) shows ozone concentrations and (**b**) shows ozone trends.

The spatial distribution and temporal variation of MDA8 ozone concentrations averaged over the study period (1–10 March) from 2019 to 2023 of those sites within the Hangzhou Bay region are shown in Figure 2. The distribution of MDA8 ozone concentrations in the Hangzhou Bay region appears relatively uniform each year, with a clear upward trend over the five years (Figure 2a). Ozone concentrations in the Hangzhou Bay area remained low during the first three years, and slightly reduced in 2021 compared to the previous two years. However, there was a significant rise in ozone concentrations starting in 2022 and a peak in 2023, following the outbreak of the earliest ozone pollution event. The areas with higher ozone concentrations each year are mainly located in Shanghai and northeastern Jiangsu Province, which indicates a stronger exposure to anthropogenic emissions in these areas (Figure 2a). Notably, while Shanghai exhibited higher ozone levels during the earlier years of low ambient concentrations, it experienced relatively lower levels in the last two years, even as surrounding regions saw rising ozone levels. This suggests that the level of ozone pollution in Shanghai was mitigated compared with other areas in Hangzhou Bay despite the early emergence of ozone pollution events here. The result shows that the trend in MDA8 ozone concentrations in Shanghai was lower than in other regions (Figure 2b). Specifically, the central and southern areas of the Hangzhou Bay region experienced the most pronounced increases in ozone concentrations from 2019 to 2023.

**Figure 3:**
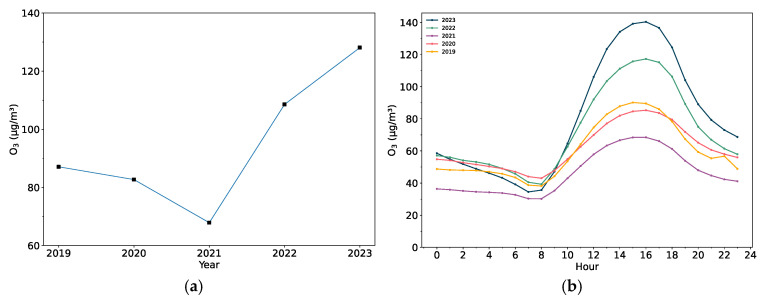
(**a**) The mean MDA8 ozone concentrations over the period from 1 to 10 March averaged for sites in the Hangzhou Bay region during 2019–2023. (**b**) Diurnal cycles of mean MDA8 ozone concentrations over the period from1 March to 10 March averaged for sites in the Hangzhou Bay region during 2019–2023.

The mean MDA8 ozone concentrations across all monitoring sites in the Hangzhou Bay region reveal a consistent downward trend in ozone concentrations in the first three years, with levels dropping below 70 μg/m^3^ in 2021. Nevertheless, a sharp increase occurred over the last two years, with concentrations far exceeding 120 μg/m^3^ (Figure 3a). The diurnal variation of mean ozone concentrations from 1 to 10 March across the Hangzhou Bay region is also shown (Figure 3b). Mean MDA8 ozone concentrations follow a similar pattern from daytime to nighttime, rising from 8 a.m. to 4 p.m., corresponding to the period of highest temperatures, and then dropping. From 2019 to 2021, the peak ozone concentrations decreased, and diurnal variations were relatively smooth, with no excessive fluctuations during these years. During 2021–2023, mean ozone levels were highly variable within one day; their levels between 4 a.m. and 8 a.m. were even as low as those of the same time in previous years. The maximum ozone concentration for one day increased considerably in the last two years, with a growth of nearly 80 μg/m^3^ compared to the lowest ozone concentration peak in 2021. In addition, there were more fluctuating diurnal variations in mean ozone concentrations and a downward trend in the minimum of mean ozone concentrations over a day from 2021 to 2023.

### 3.2. Effects of NO_x_ and VOCs on MDA8 Ozone

**Figure 4:**
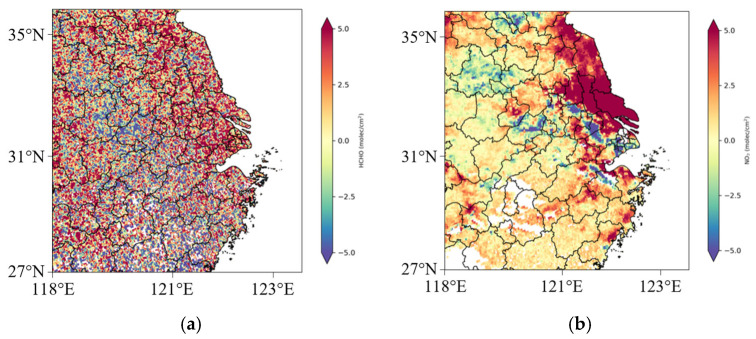

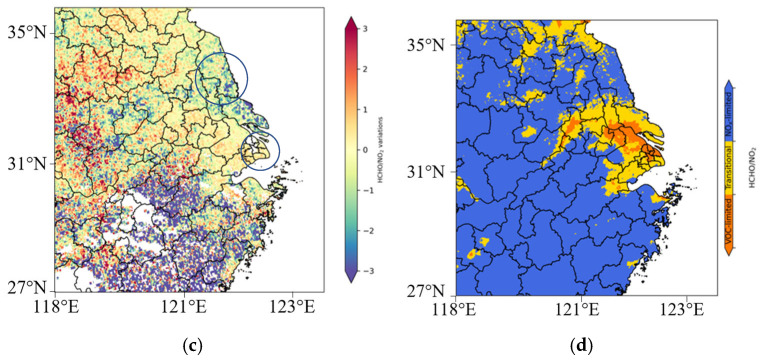
The difference in (**a**) HCHO average, (**b**) NO_2_ average, and (**c**) HCHO average/NO_2_ average during 1–10 March between 2023 and 2019. (**d**) The distribution of the ozone–NO_x_–VOC sensitivity classification in the Hangzhou Bay region during April–September 2022 was categorized by three ranges of HCHO average/NO_2_ average thresholds (<2.2, 2.2–3.3, >3.3).

The influence of VOCs and NO_x_ on this earliest ozone pollution event can be inferred from the concentrations of HCHO and NO_2_ in the region during the study period. Variations in concentrations of these two precursors up to 2023 are illustrated by calculating their concentration differences between 2023 and 2019 respectively (Figure 4). The concentrations of the two precursors in 2019 are treated as a baseline to highlight the changes observed in 2023. Although HCHO concentrations exhibit both positive and negative variations across the region, the concentrations in 2023 are still predominantly increasing compared to 2019, on the whole (Figure 4a). The maximum variation of HCHO concentrations is widespread in the whole area. The most significant increases of NO_2_ concentrations occur in Jiangsu Province (Figure 4b). Nevertheless, the extensive intensification of NO_2_ concentrations in northeastern Jiangsu Province is a key contributor to the overall increase in NO_2_ concentrations across the YRD area from 2019 to 2023. Unlike the widespread largest changes in HCHO concentrations, the most substantial rises in NO_2_ concentrations are concentrated solely in northeastern Jiangsu Province, where the magnitude of variations exceeds that of other areas.

Due to the non-linearity of ozone–NO_x_–VOC sensitivity, the rises in HCHO and NO_2_ concentrations cannot fully explain the elevated ozone concentrations in the Hangzhou Bay region. In this case, the direct column ratio between HCHO and NO_2_ and the specified HCHO/NO_2_ thresholds for the Hangzhou Bay region are also applied to the analysis of ozone pollution formation. The threshold values of HCHO/NO_2_ for the Hangzhou Bay were 2.2 and 3.3, which can be used to divide the region into three ranges: less than 2.2 (NO_x_-limited), between 2.2 and 3.3 (transitional), and greater than 3.3 (VOC-limited) [20]. The spatial distribution of the difference in HCHO/NO_2_ ratios in 2023 and 2019 is depicted in Figure 4c. The areas of Shanghai and northeastern Jiangsu Province, where higher tropospheric MDA8 ozone concentrations were recorded, are marked by blue circles in Figure 4c. In Shanghai, the variation in the HCHO/NO_2_ ratio is positive, while northeastern Jiangsu Province presents negative variations.

Based on the HCHO/NO_2_ thresholds (2.2–3.3) for the Shanghai–Jiangsu–Zhejiang–Anhui region derived from prior studies [20], the mechanism of ozone pollution formation in the Hangzhou Bay area can be more precisely identified. In the Hangzhou Bay region, areas with an HCHO/NO_2_ ratio below 2.2 are classified as VOC-limited. This indicates that reducing NO_x_ emissions or increasing VOC emissions would lead to higher tropospheric ozone levels due to excessive NO_x_. Conversely, areas with an HCHO/NO_2_ ratio above 3.3 are NO_x_-limited, meaning that reducing VOC emissions or increasing NO_x_ emissions would enhance ozone formation because of the relatively excessive VOC concentrations.

To recognize the scenarios of those areas within the Hangzhou Bay region, the distribution of the ozone formation regimes from April to September 2022 can serve as a reference for the analysis of the earliest ozone pollution event in 2023 (Figure 4d). A noted and relatively concentrated VOC-limited scenario is found in northern Zhejiang Province and northern Shanghai. The NO_x_-limited scenario dominates most other areas in the YRD, while the transitional scenario is located at the junction between NO_x_-limited regions and VOC-limited regions and in the northeastern part of the Hangzhou Bay area.

Both precursor concentrations have generally increased in Jiangsu Province, where significant ozone concentration rises are noted, and in Shanghai, which also shows relatively high ozone levels (Figure 4a,b). The HCHO/NO_2_ ratio in Shanghai has increased, that is, the concentration of HCHO rises more than that of NO_2_, placing Shanghai in the VOC-limited scenario. This directly correlates with the high ozone concentrations in the area. The HCHO/NO_2_ ratio in Jiangsu Province declines slightly, indicating that the concentration of HCHO increases slightly less than that of NO₂, but this region also experiences an annual growth in ozone concentrations since it follows the NO_x_-limited scenario (Figure 4c,d). Similar HCHO/NO_2_ ratio variations and NO_x_-limited scenarios are also available for the southern Hangzhou Bay region, corresponding to the sharp growth of MDA8 ozone concentrations from 2019 to 2023 (Figure 2b).

### 3.3. Effects of Atmospheric Conditions on MDA8 Ozone

**Figure 5:**
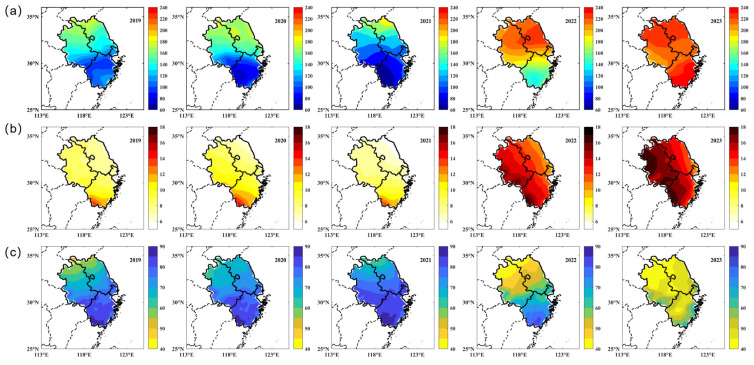
The average of (**a**) surface downward shortwave radiation flux, (**b**) air temperature (at 1000 hPa), and (**c**) relative humidity over the period from 1 to 10 March during 2019–2023 in Hangzhou Bay.

The potential meteorological impacts on the earliest ozone pollution event in 2023 could be analyzed based on atmospheric conditions from 1 to 10 March, 2023. Furthermore, the comparison of atmospheric conditions during this period across the years 2019 to 2023 will also highlight the obvious variations compared with previous years. Using the highly precise ERA5 reanalysis datasets, three relevant atmospheric variables are selected to reveal the atmospheric conditions during the study period: surface downward shortwave radiation flux, air temperature (at 1000 hPa), and relative humidity. The results present their spatial distributions in the Hangzhou Bay region averaged between 1 March and 10 March for each year between 2019 and 2023 (Figure 5).

The distribution and trend of the mean surface downward solar radiation flux were quite similar to that of the MDA8 ozone concentration from 2019 to 2023, and it also underwent a valley in 2021 and a peak in 2023 (Figure 5a). In 2023, the maximum of the mean surface downward solar radiation flux (240 W/m^2^) occurred in the southern Hangzhou Bay region, which also exhibited the most dramatic increase compared to the previous years. There was almost no significant change in the average air temperature at 1000 hPa from 2019 to 2021, either numerically or spatially distributed (Figure 5b). Its explosive growth did not appear until 2022 and reached its maximum in 2023. However, those higher air temperatures up to 18 °C were not observed in Jiangsu Province and Shanghai, as higher ozone concentrations were, but were observed in the western Hangzhou Bay region. The distribution of the average relative humidity in the first three years was relatively consistent, and it remained at high levels (>60%) in each year (Figure 5c). The overall relative humidity in the Hangzhou Bay region started to drop sharply in 2022, and there were minimum values of relative humidity (40%) in the northern Hangzhou Bay region. By 2023, the relative humidity in the Hangzhou Bay region had declined overall compared to the previous years and remained at a lower level (<60%). Overall, an atmospheric environment of high temperature, low humidity, and high solar radiation has developed in Hangzhou Bay in recent years, exacerbating the photochemical reactions leading to near-surface ozone formation.

### 3.4. Meteorological and Anthropogenic Contributions to MDA8 Ozone by Quantitative Models

MLR is proposed to quantify the meteorological and anthropogenic MDA8 ozone trends in Hangzhou Bay. Results show that the observed yearly ozone trend in early spring is 9.13 μg/m^3^ a^−1^, where weather patterns’ trend is 8.97 μg/m^3^ a^−1^, and 0.16 μg/m^3^ a^−1^ for anthropogenic activities. In other words, meteorological factors contribute largely to the early detection of ozone pollution, but anthropogenic factors are extremely low.

However, the residuals of the MLR do not match the normal distribution. Thus, we aim to identify the effect of early spring meteorology on ozone pollution by the SHAP method. Before using it, we apply the LightGBM as an interpreter to fit weather patterns. For the training set (70% of the original data), the mean absolute error (MAE) is 0.02, the mean standard error (MSE) is 0.0032, the root mean standard error (RMSE) is 0.056, and the R^2^ is 0.99. The remaining 30% of the data is the test set, and R^2^ = 0.98, RMSE = 0.16, MAE = 0.070, and MSE = 0.026.

**Figure 6:**
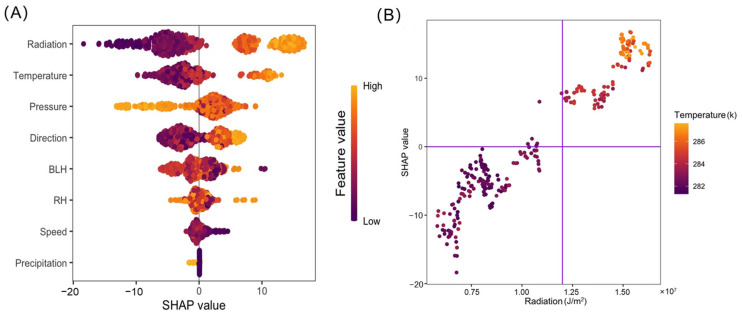
SHAP summary plot of feature importance (**A**). The SHAP dependent plot between temperature and radiation (**B**). The purple vertical line in (**B**) indicates radiation of 12 million J/m^2^.

Figure 6 depicts the SHAP summary values of weather patterns and a partial dependent plot of radiation and temperature. The mean absolute SHAP value of radiation is 7.94, followed by temperature (4.27), pressure (3.98), wind direction (3.51), BLH (2.22), RH (1.02), wind speed (0.82), and precipitation (0.14). Thus, solar radiation and air temperature lead to ozone pollution in the early spring. Relative humidity and precipitation positively affect ozone pollution, while wind speed, wind direction, pressure, and BLH have a negative effect. Based on the MLR, radiation’s variance inflation factor (VIF) is greater than 10, indicating multicollinearity between radiation and other meteorological factors. We use the SHAP technique to show that there is a high interaction effect between temperature and radiation. Their partial dependent plot further reveals that when solar radiation is beyond 12 million J/m^2^, the temperature is high, thus causing more serious ozone pollution.

## 4. Discussion

This study concludes that early spring ozone pollution in Hangzhou Bay is mainly due to meteorological factors. The observed yearly ozone trend in early spring is 9.13 μg/m^3^ a^−1^, where weather patterns contribute largely (8.97 μg/m^3^ a^−1^). More solar radiation and higher temperatures exacerbate the photochemical reactions associated with tropospheric MDA8 ozone formation, significantly when radiation exceeds 12 million J/m^2^ and high temperatures are present. Lower relative humidity also leads to increased ozone pollution. In response to anthropogenic factors, the precursors of ozone pollution, HCHO and NO_x_, show an increasing trend. According to the HCHO/NO_2_ values, the NO_x_-limited area covers most of the YRD region. At the same time, the VOC transition regime is located at the junction of the NO_x_-limited and VOC-limited areas and in the northeastern part of the Hangzhou Bay area, including Shanghai and northern Zhejiang Province.

Ozone pollution in Hangzhou Bay is becoming increasingly severe, and the areas with higher ozone concentrations each year are mainly located in Shanghai and central Jiangsu Province. We tracked the average MDA8 ozone concentrations at all monitoring sites in the Hangzhou Bay area between 1 and 10 March from 2019 to 2023. Ozone concentrations continued to decline, even below 70 μg/m^3^ in 2021, then increased sharply and are now well above 120 μg/m^3^. The diurnal fluctuations in mean ozone concentrations varied considerably during 2021–2023, with a decreasing trend in the one-day minimum of mean ozone concentrations.

Hangzhou Bay ozone appears to have been polluted earlier, especially in Jiaxing. Compared with the initial two years, when ozone pollution occurred late and pollutant concentrations were high, the earliest ozone pollution events in the latter three years of were characterized by early onset and relatively low pollutant concentrations. We reveal the formation factors using sensitivity zoning and meteorological factors for the early onset of ozone pollution in Hangzhou Bay. YRD is mainly located in the NO_x_-limited regime, indicating the cost-effectiveness of NO_x_ reduction. The transitional scenario is located at the junction of the NO_x_-limited and VOC-limited regimes and in the northeastern part of the Hangzhou Bay area. Shanghai and northern Hangzhou Bay follow the VOC-limited scenario, a direct explanation for its high ozone levels.

For meteorological conditions, there are significant variations in all three atmospheric variables from 2022 to 2023 compared to previous years throughout the whole Hangzhou Bay region, resulting in an atmospheric environment with high temperature and low humidity here. In this case, sufficient solar radiation and higher air temperatures will exacerbate the photochemical reactions associated with the formation of tropospheric ozone [32]. In addition, a warm and dry atmospheric environment typically indicates an anticyclonic system [1,4]. This meteorological system would produce a stable atmospheric condition that weakens the diffusion of VOCs and NO_x_, prolonging the photochemical reaction time between them and thus generating more tropospheric ozone [12]. Lower relative humidity could reduce the amount of precipitation, which would not be favorable for the dissolution and deposition of atmospheric pollutants including tropospheric ozone and its precursors, thus contributing to higher concentrations of them [33]. As warmer and drier atmospheric conditions intensify, some plants will release biogenic VOCs, an essential source of tropospheric ozone precursors, for longer due to the moisture shortage. High levels of biogenic VOC emissions, previously restricted to regular seasons such as summer, would persist for longer periods, which might lead to an earlier onset of ozone pollution events [11].

Compared to the formation mechanism in Hangzhou Bay during the warm season, the pollution in early spring is still located in the VOC-limited and transitional regimes [29,34]. However, NO_x_-limited regimes dominate the YRD [30]. Moreover, early spring pollution may be more related to meteorological factors, notably solar radiation and air temperature [35]. However, this study mainly relied on quantitative methods, and future studies could further explain the formation mechanism of ozone spring pollution using chemical transport models. Our study combines multiple quantitative models to reveal the drivers of early onset of ozone pollution in Hangzhou Bay, thus providing scientific guidance to mitigate ozone pollution.

## 5. Conclusions

The Hangzhou Bay area is home to one of the seven largest petrochemical agglomerations in China, with worsening ozone pollution and an early onset of ozone pollution on 7 March 2023 (Jiaxing). Among the anthropogenic factors of ozone pollution, the YRD region is mainly in the NO_x_-limited regime, such as in northeastern Jiangsu Province, indicating that NO_x_ control effectively reduces ozone. The Hangzhou Bay region, including Shanghai and northern Zhejiang Province, is mainly located in the VOC-limited regime, hence reducing VOCs will be more effective in mitigating ozone pollution here. Our results show that HCHO column concentrations have been increasing in VOC-limited regions and NO_x_ column concentrations have been increasing more quickly than HCHO in NO_x_-limited regions between 1 and 10 March of each year from 2019 to 2023. Based on quantitative models and ERA5 reanalysis data, high temperature, low humidity, and high radiation lead to increased ozone pollution, indicating the vital role of meteorological factors in ozone pollution in the context of climate change.

## Figures and Tables

**Table 1:** Earliest dates when MDA8 ozone concentrations exceeded the national environmental air quality daily average standard secondary limitation (160 μg/m^3^) among seven major cities (Shanghai, Hangzhou, Ningbo, Jiaxing, Huzhou, Shaoxing, and Zhoushan) in the Hangzhou Bay region from 2015 to 2023.

Year	Earliest Date	City	MDA8 $O_{3}$ Concentration (μg/m^3^)
2015	10 April	Huzhou	184.7
2016	13 April	Huzhou	192.0
2017	4 April	Huzhou	163.7
2018	28 March	Jiaxing	178.3
2019	17 March	Shaoxing	173.0
2020	18 March	Shanghai	164.0
2021	29 March	Huzhou	162.0
2022	15 March	Hangzhou	164.0
2023	7 March	Jiaxing	171.0

The criterion for identifying an ozone pollution event is that the concentration of MDA8 ozone in a city on a given day exceeds 160 μg/m^3^, the baseline of the national environmental air quality daily average standard secondary limitation.

## Data Availability

The TROPOMI data for NO_2_ and HCHO are available from the Earth Engine Data Catalog at (https://developers.google.com/s/results?q=Sentinel-5P%20TROPOMI%20observations%20&text=Sentinel-5P%20TROPOMI%20observations, accessed on 10 May 2023), the MDA8 ozone concentration was calculated based on ozone 8 h datasets from the National Urban Air Quality Real-Time Publishing Platform at https://air.cnemc.cn:18007/ (accessed on 15 April 2023). Radiation, temperature, and other meteorological data are available from ERA5 reanalysis data at https://cds.climate.copernicus.eu (accessed on 4 May 2023).
